# Novel bias-reduced coherence measure for EEG-based speech tracking in listeners with hearing impairment

**DOI:** 10.3389/fnins.2024.1415397

**Published:** 2024-11-06

**Authors:** Oskar Keding, Emina Alickovic, Martin A. Skoglund, Maria Sandsten

**Affiliations:** ^1^Centre for Mathematical Sciences, Lund University, Lund, Sweden; ^2^Eriksholm Research Centre, Oticon A/S, Snekkersten, Denmark; ^3^Department of Electrical Engineering, Linköping University, Linköping, Sweden

**Keywords:** coherence, EEG, neural speech tracking, auditory attention, multitapers, hearing impairment

## Abstract

In the literature, auditory attention is explored through neural speech tracking, primarily entailing modeling and analyzing electroencephalography (EEG) responses to natural speech via linear filtering. Our study takes a novel approach, introducing an enhanced coherence estimation technique to assess the strength of neural speech tracking. This enables effective discrimination between attended and ignored speech. To mitigate the impact of colored noise in EEG, we address two biases–overall coherence-level bias and spectral peak-shifting bias. In a listening study involving 32 participants with hearing impairment, tasked with attending to competing talkers in background noise, our coherence-based method effectively discerns EEG representations of attended and ignored speech. We comprehensively analyze frequency bands, individual frequencies, and EEG channels. Frequency bands of importance are shown to be delta, theta and alpha, and the important EEG channels are the central. Lastly, we showcase coherence differences across different noise reduction settings implemented in hearing aids (HAs), underscoring our method's potential to objectively assess auditory attention and enhance HA efficacy.

## 1 Introduction

Listening is a biological process that engages the entire auditory system. The process, from sound pressures to neural activations, includes (non-linear) transforms of the peripheral auditory system as well as complex processing within the central auditory system. The listening process also affects the electrical activity of the brain, which can be measured on scalp-level using electroencephalography (EEG). Linear filter estimation, referred to as temporal response functions (TRFs) (Crosse et al., 2016; Alickovic et al., 2019; Geirnaert et al., 2021), has been shown to capture the relations between the auditory system and EEG signatures (O'sullivan et al., 2015). State-of-the art methods employ TRFs to analyze phonemes (Di Liberto et al., 2015; Brodbeck et al., 2018; Carta et al., 2023) and semantic content (Broderick et al., 2018; Gillis et al., 2021). The TRF methods are also particularly valuable for decoding auditory attention, specifically in identifying the attended talker from EEG signals in challenging listening situations like cocktail-party environments (Cherry, 1953). Effectively decoding auditory attention to distinguish and enhance attended speech in multi-talker situations holds significance for hearing aid (HA) applications (Lunner et al., 2020; Alickovic et al., 2020, 2021; Andersen et al., 2021).

The mentioned studies, along with others, provide evidence that neural speech processing exhibits a linear component. Specifically, there are robust linear relationships between the speech envelope and the concurrent EEG signatures. Hence, spectral coherence analysis between the speech envelope and EEG signals can confidently identify and analyze the underlying system to a significant degree, as demonstrated in Viswanathan et al. (2019) and Vander Ghinst et al. (2021). Spectral coherence is also a measure of the linear coupling between two signals, calculated as the cross-spectrum normalized by the respective auto-spectrum of both signals.

Various methods exist for estimating spectral coherence, where the Welch method is the most commonly applied. In this approach, uncorrelated Fourier-based spectrum estimates from multiple data segments (that may overlap) are averaged (Welch, 1967). Another state-of-the-art approach is Thomson's multitaper method, which multiplies data with various tapering windows before Fourier analysis and subsequent averaging (Thomson, 1982; Walden, 2000; Karnik et al., 2022). Multitapers are often applied in various applications for robust spectral estimation of EEG (Alickovic et al., 2023; Reinhold and Sandsten, 2022; Viswanathan et al., 2019; Babadi and Brown, 2014; Hansson-Sandsten, 2010).

The statistics in coherence estimation using Welch method (Carter and Nuttall, 1972; Carter et al., 1973) as well as the Thomson's multitaper method (Thomson, 1982; Bronez, 1992; McCoy et al., 1998; Brynolfsson and Hansson-Sandsten, 2014; Hansson-Sandsten, 2011) has been thoroughly studied. The coherence of 1 signifies a perfect linear connection between signals, while no coupling yields the ideal coherence is 0. In practice, the lack of infinite data always introduces a significant bias upwards toward positive values in the no coupling case (Carter and Nuttall, 1972). In comparison, the variance can be assumed to be small when a reasonable amount of data is available (Carter et al., 1973; Thomson, 1982).

This paper introduces a new method for EEG-speech envelope coherence estimation, denoted bias-reduced coherence (BRC). The method aims to decrease the bias of estimation, while assessing the effectiveness of coherence in analyzing the relationship between speech envelope and EEG responses. With a certain choice in the cross-spectra estimation, as part of the coherence measure, the bias at low linear coupling can be reduced compared to traditional methods. Considering that EEG responses are susceptible to random noise, any detected linear coupling is expected to be weak. Real data is employed to evaluate the coherence methods and showcase their potential in decoding auditory attention. Furthermore, since coherence is applied to signals influenced by 1/*f* spectrally shaped noise prevalent in EEG data (Bénar et al., 2019), this study also investigates biases arising from this phenomenon. Coherence “levels out” the slanted noise in the coherence spectrum, and peaks that are widened by the taper kernel will have a bias toward higher frequencies. This paper quantitatively analyzes this bias and its implications for EEG and hearing application. The study's hypotheses are as follows.

### Two types of anticipated biases in speech-EEG coherence estimation

1. Due to the high level of noise present in EEG data, we anticipate an overall upward bias across all frequencies from all methods. We propose that the new BRC can mitigate this bias, due to including taper phase information.
2. We expect coherence peaks from all coherence estimates to shift toward higher frequencies, due to the slanted nature of EEG noise. The extent and nature of this shift is hypothesized to be dependent on factors such as the number of tapers, data length, and the shape and power of the noise present in signals.

### Auditory attention and hearing aid effects manifested in speech-EEG coherence changes

1. Differences in speech-EEG coherence between attended and ignored speech, specifically within the delta, theta, and alpha frequency bands associated with auditory processing, are anticipated when applying these methods to a population using HAs. For these bands, we expect clearer distinctions in coherence estimates with the BRC, since this improves bias and variance of coherence estimates.
2. Speech-EEG coherence differences are expected to be larger when HA noise reduction feature is activated compared to when it is deactivated.

This work extends our previous study (Keding et al., 2023), which introduces the novel BRC method that utilizes more of the phase information from tapered data segments. In Keding et al. (2023) the case when coherent signals are affected heavily by noise (causing low expected true coherence levels) is analyzed. Also, the base-level biases are compared for the traditional and the new BRC method, showing lower bias for BRC. Here, we present further analytical analysis of aspects evident during application of coherence as a measure within EEG analysis, focusing on neural speech tracking. Further comparisons of coherence methods topographically over channels are provided. Additionally, we investigate coherence as a metric for objectively assessing the effect of HAs on auditory attention during listening tasks.

The paper is structured as follows. Firstly, the traditional coherence method and the BRC are presented in Section 2, where the reduction in upward magnitude bias of BRC is explained. Analysis of the positional bias of peaks in the coherence spectrum due to 1/*f* noise is evaluated analytically as well as in simulations. Section 3 outlines the experimental design, pre-processing of data and statistical methods used in real data analysis. Results on real data are presented in Section 4, with related discussion in Section 5, highlighting the capabilities and limitations of coherence methods in auditory attention decoding and in objective evaluation of HA benefits. Conclusions are found in Section 6.

## 2 Coherence estimation

Spectral coherence *C*_*xy*_(*f*) is the measure of the magnitude of a linear coupling between two signals in a system, ranging 0 ≤ *C*_*xy*_(*f*) ≤ 1, here defined by its squared form


(1) $$\begin{array}{l} C_{xy}\left(f\right)=\frac{S_{xy}{\left(f\right)}^{2}}{S_{xx}\left(f\right)S_{yy}\left(f\right)} \end{array}$$

*S*_*xy*_(*f*) is the cross-amplitude spectrum between signals *x*(*n*) and *y*(*n*). *S*_*xx*_(*f*) and *S*_*yy*_(*f*) are the respective auto-spectra. Coherence, as denoted in Equation 1, is referred to as Magnitude Squared Coherence (MSC).

### 2.1 Magnitude squared coherence

Estimating the MSC entails estimating the different spectra according to


(2) $${\hat{C}}_{xy}(f)=\frac{{\hat{S}}_{xy}{\left(f\right)}^{2}}{{\hat{S}}_{xx}(f){\hat{S}}_{yy}(f)}$$

The auto-spectra are estimated through averaging sub-spectra for each pair of L number of data segments and K number of data tapers as


(2) $${\hat{S}}_{xx}(f)=\frac{1}{KL}\sum_{k=1}^{K}\sum_{l=1}^{L}X_{k,l}(f)X_{k,l}{\left(f\right)}^{*}$$

(4) $${\hat{S}}_{yy}(f)=\frac{1}{KL}\sum_{k=1}^{K}\sum_{l=1}^{L}Y_{k,l}(f)Y_{k,l}{\left(f\right)}^{*}$$

where Fourier transforms of the *l*:th data segments *x*_*l*_(*n*), *y*_*l*_(*n*) over time samples *n* and total lengths *N*, and *k*:th tapering window *h*_*k*_(*t*) of respective signal are


(5) $$\begin{array}{l} X_{k,l}\left(f\right)=\overset{N-1}{\underset{n=0}{\sum }}x_{l}\left(n\right)h_{k}\left(n\right)e^{-i2\pi fn} \end{array}$$

(6) $$\begin{array}{l} \;Y_{k,l}\left(f\right)=\overset{N-1}{\underset{n=0}{\sum }}y_{l}\left(n\right)h_{k}\left(n\right)e^{-i2\pi fn} \end{array}$$

where *i* is the imaginary unit.

There are multiple options when constructing the cross-spectra from sub-spectra. Here we will consider two such options. The first has been used in Viswanathan et al. (2019) in speech envelope to EEG coherence estimation


(7) $${\hat{S}}_{xy}^{TRAD}(f)=\frac{1}{KL}\sum_{k=1}^{K}|\sum_{l=1}^{L}X_{k,l}(f)Y_{k,l}{\left(f\right)}^{*}|$$

Coherence estimation using this method is denoted the *traditional method*. In Equation 7 and from now on in the paper, the *L* data segments are assumed non-overlapping. A second option was introduced in our previous work (Keding et al., 2023), where both sums are taken before the absolute value as


(8) $${\hat{S}}_{xy}^{BRC}(f)=\frac{1}{KL}|\sum_{k=1}^{K}\sum_{l=1}^{L}X_{k,l}(f)Y_{k,l}{\left(f\right)}^{*}|$$

Note that these two options gives the same auto-spectra, since all sub-spectra are real and positive. The resulting estimator of coherence with ${\hat{S}}_{xy}^{BRC}(f)$ is shown to decrease the bias upwards in magnitude for low coherence scenarios. This due to the traditional method averaging power over tapers without taking the phase information of tapers into account, which BRC does. This is shown in Keding et al. (2023). Coherence estimation using this method is therefore denoted *bias-reduced coherence* (BRC). The method in Equation 8 utilizes the phase information of the signals in estimation of the final cross-spectrum. A third option where the order of sums in Equation 7 is reversed, so absolute values of the sum of tapers are taken, is not considered here as *L*>*K* in most applications.

**Figure 1:**
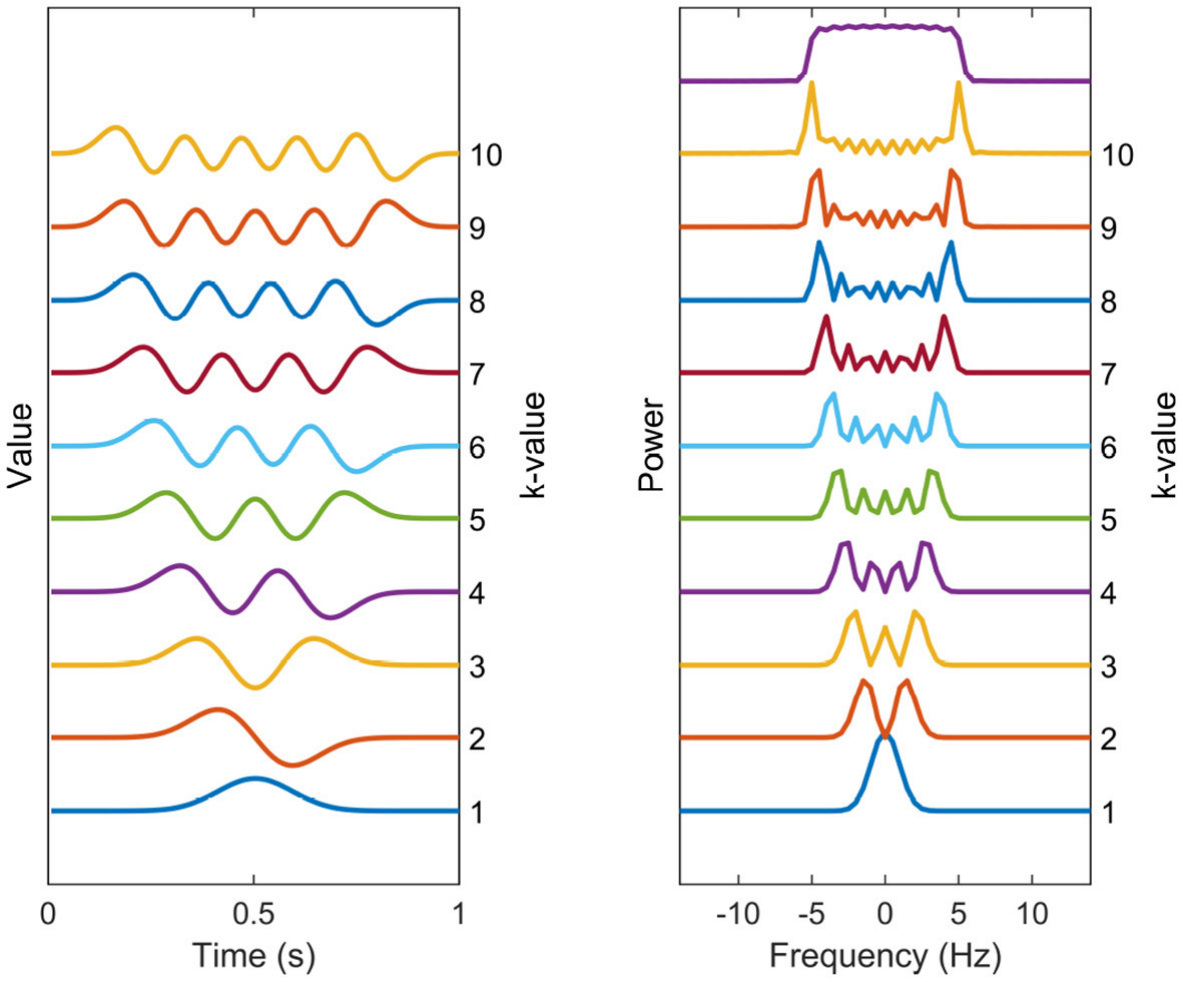
Spectral estimation windows. The temporal representation and power spectra of each of the 10 first DPSS windows used in the multitaper coherence estimation. The tapers are enumerated from bottom to top. The sum of the taper kernels is shown above the separate spectral kernels, effectively showing the width of the narrowband leakage effects.

A common choice, in accordance with the Thomson's multitaper method, for the set of tapering windows {_*h*_*k*_(*t*)}1, ..., *K*_ are the Discrete Prolate Spheroidal Sequences (DPSS), which can be seen in time and frequency domain in Figure 1 (Slepian, 1978). These are the windows that maximize the main lobe (or within-band) power relative to broadband power for a normalized frequency bandwidth *W*, but have no closed-form expression. They have been shown to have effective variance-reduction characteristics. Although it is argued here that these are suitable choices for tapering windows, the tapering windows can be chosen from another set, with the general methodology staying identical.

The number of DPSS windows theoretically can be infinite, but using a large amount of tapering windows is not advisable due to its potential to significantly increase the narrowband leakage. This leads to the smearing of the spectrum, making spectral peaks of neighboring frequencies to be indistinguishable from one another. This phenomenon become particularly problematic when estimating coherence in 1/*f*-shaped noise, as discussed in Section 2.2. Instead, a practical approach involves selecting the desired bandwidth of the tapering windows according to the specific application and limit the number of windows accordingly. The DPSS windows are defined in terms of both their shape and the number of windows required to achieve this bandwidth. A common choice for the number of windows is *K* = 2*NW*, where *N* is the lengths of signals and *W* is the normalized frequency bandwidth.

A overall bias analysis of the traditional coherence estimation technique, compared to the BRC has been given in Keding et al. (2023). In this work, the BRC was shown to significantly reduce the bias of the coherence estimate level when there is no phase-locking between signals in channel *x* and *y*. The traditional method wrongly shows a peak in the coherence spectrum even though there is no phase-locking present. If 1/*f*-shaped noise is added to the phase-locked signal in either or both channels, the peaks of coherence for narrowband signals are also spectrally shifted toward higher frequencies. This simple observation made in Keding et al. (2023) is further expanded and analyzed in the following section.

### 2.2 Spectral positional bias in EEG-like noise

In various EEG-related applications, coherence measures are employed to analyze coherence spectra between EEG channels, or between EEG channels and sensory stimuli. This analysis allows the detection of key frequencies showing notable correlation. The identification of these specific correlation frequencies finds applications in tasks like filtering, denoising, and drawing functional insights about the brain.

However, a common challenge faced in visually inspecting the coherence spectra, or any coherence method based on the Fourier transform, is to identify key frequencies in the correlation between channels in the presence of unwanted spectral shifting of relevant coherence energy peaks. This shift is attributed to the presence of slanted 1/*f* noise, indicative of irrelevant brain activity. As the coherence normalizes the base levels of spectrum, the 1/*f* pattern becomes flattened, making it difficult to perceive its impact during peak analysis. This effect is particularly pronounced at lower frequencies, where the 1/*f* noise spectrum exhibits the most prominent degree of gradient.

#### 2.2.1 Approximate coherence for sinusoidal signal

To illustrate the positional bias of peaks in the coherence spectrum when one or more signals are disturbed by 1/*f* noise, we introduce an expression for the coherence of a single frequency coupled model. Although the positional bias impacts any peaked spectra similarly, a simple case showing the bias involves single frequency oscillations is:


(9) $$\begin{array}{l} x\left(n\right)=s\left(n\right),\;y\left(n\right)=s\left(n\right)+\sigma_{p}e_{p}\left(n\right) \end{array}$$

where the signal $s\left(n\right)=F_{f\to n}\left[\delta_{f_{0}}\left(f\right)\right]$ represents a complex sinusoidal and the noise has a spectral distribution $E\left[|F_{n\to f}\left[e_{p}\left(n\right)\right]{|}^{2}\right]=1/f^{\alpha }$, α>0.

In this subsection, it is assumed that $H_{tot}\left(f\right)=\overset{K}{\underset{k=1}{\sum }}|H_{k}\left(f\right){|}^{2}=\text{rect}\left(f/B\right)$, the combined taper kernel, is a box function of energy one and frequency range of *B* = 2*Wf*_*s*_, as shown in Thomson (1982), where *f*_*s*_ denotes the sampling frequency. The expected value of MSC between *x* and *y* can be approximated by the ratio of smeared cross-spectra and smeared auto-spectra of signals, since these are the expectations of the cross/auto-spectra. This gives a fairly simple expression for the final expectation of the coherence estimate. For *f*>*B*/2, an initial application of a zero:th order Taylor expansion of the expectation yields


(10) $$E[{\hat{C}}_{xy}(f)]\approx \frac{|E[{\hat{S}}_{xy}(f)]{|}^{2}}{E[{\hat{S}}_{xx}(f)]E[{\hat{S}}_{yy}(f)]}$$

(11) $$\begin{array}{l} =\frac{|E\left[S_{xy}\left(f\right)\right]*H_{tot}\left(f\right){|}^{2}}{\left(E\left[S_{xx}\left(f\right)\right]*H_{tot}\left(f\right)\right)\left(E\left[S_{yy}\left(f\right)\right]*H_{tot}\left(f\right)\right)} \end{array}$$

(12) $$\begin{array}{l} =\frac{|\delta_{f_{0}}\left(f\right)*H_{tot}\left(f\right){|}^{2}}{\left(\delta_{f_{0}}\left(f\right)*H_{tot}\left(f\right)\right)\left(\left(\delta_{f_{0}}\left(f\right)+\frac{\sigma_{p}^{2}}{f^{\alpha }}\right)*H_{tot}\left(f\right)\right)} \end{array}$$

(13) $$\begin{array}{l} =\frac{H_{tot}\left(f-f_{0}\right)}{H_{tot}\left(f-f_{0}\right)+\frac{\sigma_{p}^{2}}{f^{\alpha }}*H_{tot}\left(f\right)} \end{array}$$

(14) $$=\{\begin{array}{ll} \frac{1}{1+\frac{\sigma_{p}^{2}}{f^{\alpha }}*H_{tot}(f)} & f\in [f_{\text{0}}-\frac{B}{\text{2}},f_{\text{0}}\text{+}\frac{B}{\text{2}}]\\ 0 & \text{ otherwise} \end{array}$$

where


(15) $$\frac{1}{f^{\alpha }}*H_{tot}(f)=\int_{-B/2}^{B/2}{\left(\nu -f\right)}^{-\alpha }d\nu =$$

(16) $$=\{\begin{array}{ll} \frac{1}{-\alpha +1}({\left(f+B/2\right)}^{-\alpha +1}-{\left(f-B/2\right)}^{-\alpha +1}) & \alpha <\text{1}\\ \log(f+B/2)-\log(f-B/2) & \alpha \text{=1}\\ \frac{1}{-\alpha +1}({\left(f+B/2\right)}^{-\alpha +1}-{\left(f-B/2\right)}^{-\alpha +1}) & \alpha >\text{1} \end{array}$$

As can be easily observed in Equation 14, the actual bias beyond the main lobe of the taper kernel is disregarded. In reality, these spectra have some estimated coherence, although lower than the main lobe coherence shown, as discussed in Keding et al. (2023). One can identify this expression as an increasing coherence spectrum within the bandwidth of the tapers used. Perhaps the most frequent choice is α = 1, which gives 1/*f* noise. A bias error in Equation 14 is expected, since the approximation is crude in reality. Additionally, an overall bias upwards is missing, that is observed in the no-coherence case, as estimation of zero-coherence is impossible with finite data. However, this does not affect the frequency shifting of peaks.

**Figure 2:**
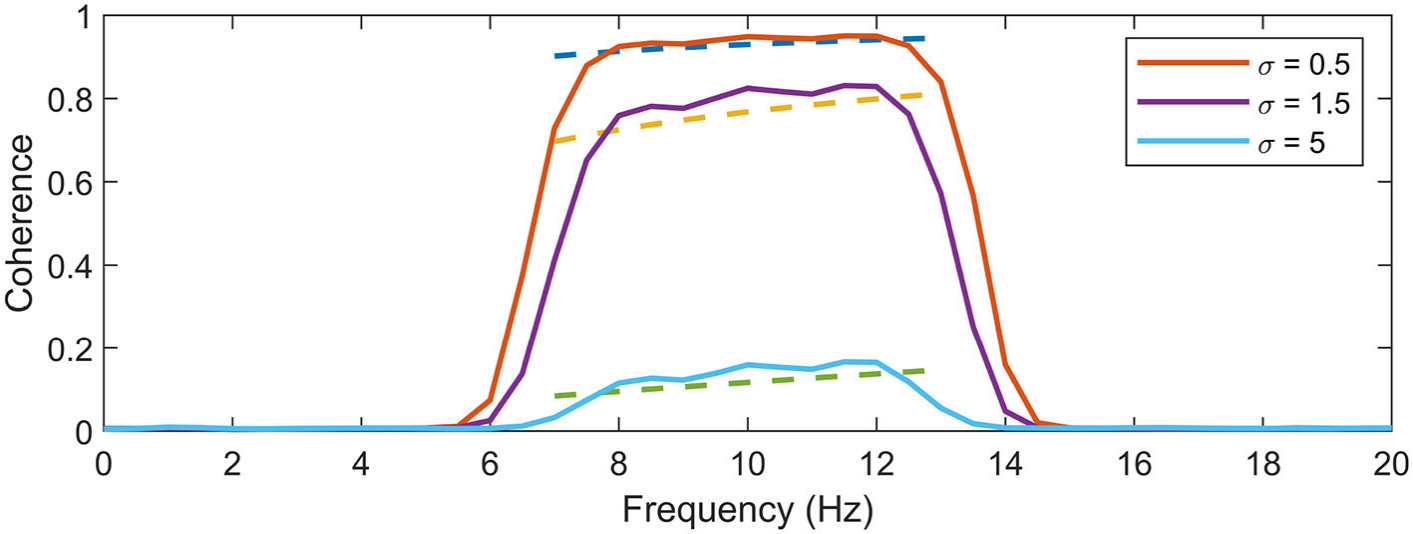
Evaluation of expectation expression. The expression for coherence expectation in dashed lines is shown along the corresponding average coherence of 10,000 estimates on a sinusoidal signal in two channels, with added 1/*f* noise in one of the channels. The comparison is made for three different noise levels, σ = [0.5, 1, 5], which corresponds the pairs of expectations in ascending order. One can see that the expression estimates the behavior of the coherence expectation very well, up to a constant. Ideal coherence lies in a single peak at *f*_0_ = 10 Hz.

Figure 2 presents a comparison between the empirical expectation of *N*_*real*_ = 10, 000 simulations of signals according to Equation 9 to the Equation 14. Signal parameters were set to *f*_0_ = 10, *N* = 128, sampling frequency *f*_*s*_ = 128, σ = [0.5, 1, 5] and α = 1, yielding a taper bandwidth *B* = 6 Hz. The simulations demonstrate the positional bias of the peak clearly in all noise levels of the slanted noise. One can see that the expression follows the expectation of the coherence estimates very well, up to a scale factor.

#### 2.2.2 Effects of signal and method parameters

Characterizing parameter effects on the frequency shifts of peaks in coherence is possible with some a priori knowledge about signals and the coherence estimation method. These shifts depend on the data segment length and the number of tapers used in a multitaper coherence estimation method.

**Figure 3:**
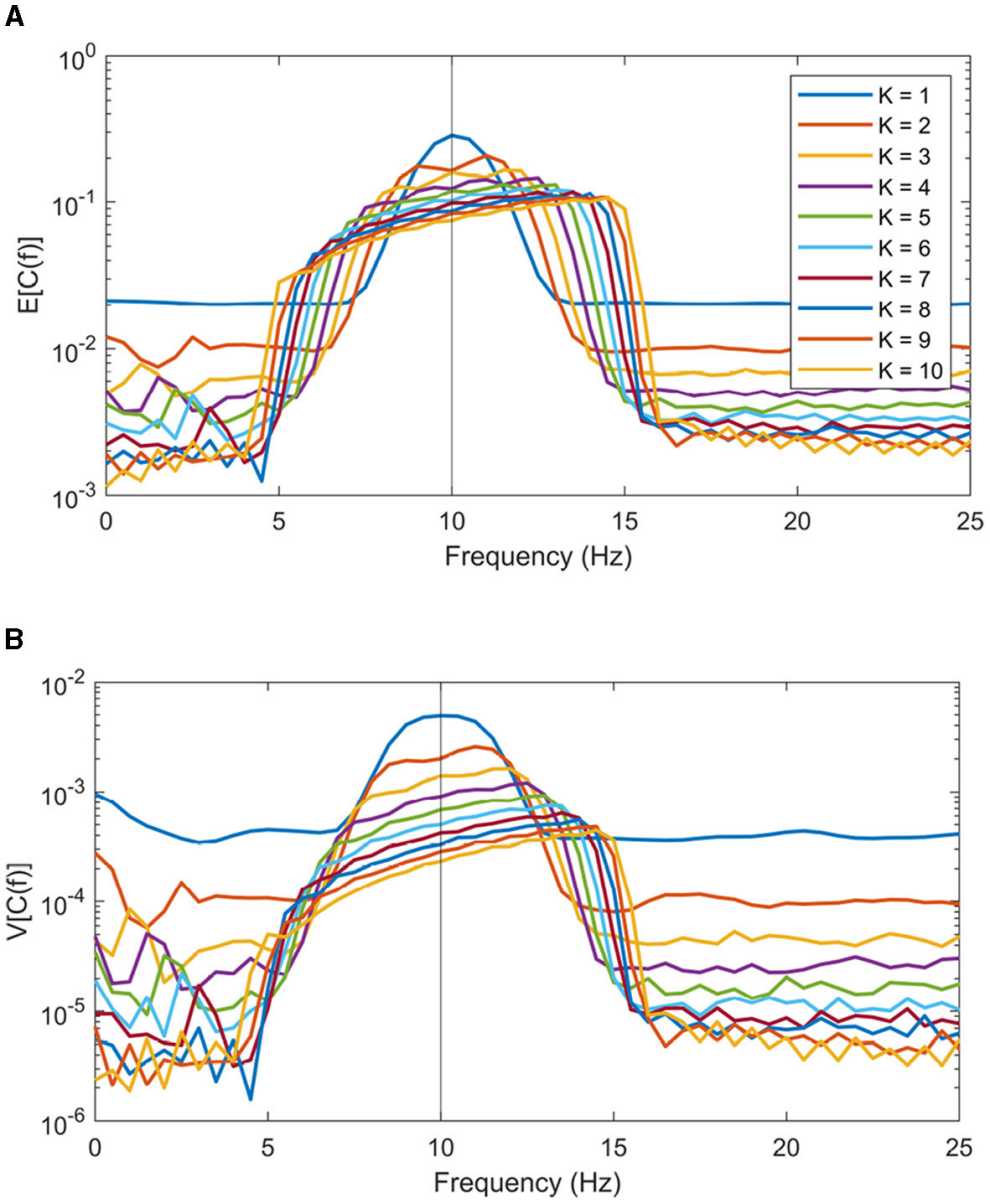
Expectation **(A)** and variance **(B)** of coherence estimation using BRC with a varying amount of tapers included. The number of tapers *K* ranges from 1 to 10.

Firstly, the data length *N* modulates the taper kernel's narrowband width. Decreasing *N* will linearly increase frequency length of the bias of coherence peaks. Secondly, introducing more tapers in a multitaper scenario will further amplify this bias, although there is a trade-off involving variance reduction with higher *K* values. Simulations are made to visualize this effect. In these simulations, signals are defined as per Equation 9, with *f*_0_ = 10 Hz. Colored noise *e*_*p*_(*n*) is made by Fourier transforming white noise and then multiplying it by the 1/*f*-spectrum before transforming back. Coherence estimates were computed using BRC on *N*_*real*_ = 10, 000 generated signals with parameters *N* = 128, *f*_*s*_ = 128, *L* = 50 and varying *K* from 1 to 10. Figures 3A, B show the empirical expectation and variance of the coherence estimates across all repetitions. These figures reveal that the coherence peak shifts more in the spectral domain as the number of included window increases. It is worth noting that method parameters, such as data lengths *N* and number of windows *K*, are typically chosen in an application and can be adjusted accordingly during analysis.

On the other hand, parameters that influence the shifting bias are not as easily quantified prior to analysis. Examples of such parameters include the noise-level of the 1/*f* noise and the spectral position of the coherence peak in question. Referring back to Equation 14, the SNR of 1/*f* noise doesn't affect how much the peaks are shifted spectrally; it only impacts the magnitude of the resulting coherence estimate. An exception occurs with very high SNR, where the coherence approaches one, making the slanted peak indistinguishable from a plateau. In this case, the risk of misinterpretation is minimal. Nevertheless, in many applications, coherence values are not substantially large.

It is notable that the true frequency of the peak does not alter how wide peaks are shifted, but that it does influence the slope of the widened band, resulting in a sharper or more blunt peak. This occurs due to the flattening of slope of the 1/*f* noise spectrum at higher frequencies. Additionally, there is often noise in the *x* channel. Parameter effects on the frequency shifting of peaks are consistent when 1/*f* noise (or white) is added to *y*, albeit with an overall lower coherence.

## 3 Experimental setup and statistical methods

The methods discussed in this study were assessed using an EEG dataset collected from individuals with hearing impairment. This dataset has been previously analyzed in several studies using different analysis approaches (Andersen et al., 2021; Keding et al., 2023). Data collection started 1st of June 2020 and ended on 7th of September 2020. Experimental protocol has been approved by the Science Ethics Committee for the Capital Region of Denmark (journal no. H20028542). The study was conducted according to the Declaration of Helsinki, and all the participants signed a written consent prior to the experiment. More information and details about data can be found in Andersen et al. (2021).

### 3.1 Participants and experimental design

The study included 31 experienced HA users, aged 65.5 years on average, with mild to moderately severe sensorineural hearing loss. All participants were native Danish speakers without a history of neurological disorders, dyslexia or diabetes mellitus. The experiment consisted of 84 trials, with the first four trials designated for training and the remaining 80 for testing and analysis. Each trial commenced with a short silence, succeeded by 5 s of background noise. Subsequently, 33 s of concurrent attended and ignored speech, along with background noise, were presented. Post-trial, participants responded to a two-choice question about the content of the attended speech (Alickovic et al., 2020, 2021; Andersen et al., 2021).

Speech stimuli were presented through a sound card (RME Hammerfall DSP multiface II, Audio AG, Germany) to six loudspeakers (Genelec 8040A; Genelec Oy, Finland). The loudspeakers were positioned at 30° (T1-T2), 112.5° (B1-B2), and 157.5° (B3-B4) azimuth. The audio was played at a 44.1 kHz sampling rate. Attended and ignored speech were presented at 73 dB SPL in T1 or T2, randomized evenly over trials. Background noise was presented at 70 dB SPL in B1-B4, with a mix of four talkers in each loudspeaker. T1 and T2 had talkers of opposite sexes presented at each trial. EEG data were recorded at 1024 Hz using a BioSemi ActiveTwo 64-channel EEG system in a 10–20 layout.

Participants wore HAs during all trials, split into four sessions of 20 trials each. These sessions were randomly ordered and had different HA noise reduction (NR) settings. Two sessions had NR turned OFF (referred to as HA NR OFF), and two sessions had NR turned ON (referred to as HA NR ON) with two different NR algorithms, as described in Andersen et al. (2021). However, these NR algorithms are not discussed in this study.

### 3.2 Preprocessing steps

The attended and ignored speech signals in each trial were decimated to a sampling rate of 256 Hz, and envelopes were calculated as the absolute value of the analytic version of speech signals, following similar studies (O'sullivan et al., 2015; Alickovic et al., 2019). The EEG data were re-referenced to the average of mastoid channels, bandpass filtered between 0.5 and 70 Hz, notch filtered to remove line noise, and resampled to 256 Hz. On average, 0.87 channels per subject were identified as bad channels and were interpolated using neighboring channels. Independent component analysis (Bell and Sejnowski, 1995) was performed to remove components not related to brain activity. On average, 17 components per subject were removed. The analysis involved two speech envelope signals and 64 EEG channels of EEG for each trial.

### 3.3 Statistical analysis

Statistical *p*-value testing assessed the significance of method choices (traditional or BRC estimation method) on differences of speech-EEG coherence between attended and ignored speech. Speech-EEG coherence estimates were computed for each trial using both the traditional method and the BRC. This was done using *L* = 33 number of data segments that are *T* = 1 *s* long, and *K* = 10 data tapers. Coherence values were then averaged across each EEG band (denoted *B*), or retained as estimates for each frequency bin. Bands are defined as delta (1–4 Hz), theta (4–8 Hz), alpha (8–12 Hz), beta (12–30 Hz), and gamma (30–128 Hz). A null hypothesis was formulated, positing that the grand average (over all subjects and trials) of attended speech coherence $C_{att}^{B}$ and ignored speech coherence $C_{ign}^{B}$ are equal. The alternative hypothesis suggests that $C_{att}^{B}$ is greater than $C_{ign}^{B}$.


$$\begin{array}{l} H_{0}:C_{att}^{B}-C_{ign}^{B}\leq 0 \end{array}$$

$$\begin{array}{l} H_{1}:C_{att}^{B}-C_{ign}^{B}>0 \end{array}$$

To estimate the null distribution, $D^{B}=C_{att}^{B}-C_{ign}^{B}$, by bootstrap sampling, the sign of *D*^*B*^ was randomized for each subject trial. Next, the mean over all trials and subjects was taken to make one grand average sample from the null hypothesis (Viswanathan et al., 2019; Te and Ap, 2002). This procedure is repeated 500,000 times to approximate the underlying null distribution. Finally, the probability of actual observed coherence grand average difference was derived from the null distribution.

## 4 Results

This section applies the BRC method and the traditional method of coherence estimation to the experimental data detailed in Sections 3.1 and 3.2. Speech-EEG coherence is shown to be useful in decoding attention in a multi-talker scenario. This is done by comparing the resulting *p*-values of the difference of coherence when *x* is either envelopes of attended or ignored speech. As we are interested in not simply the significance of results, but the overall sensitivity of the novel method BRC compared to the traditional method, comparing *p*-values illuminates in this aspect. Smaller values of *p*-values indicate found larger differences between attended speech and ignored speech, which indicate a more sensitive model in this context.

**Figure 4:**
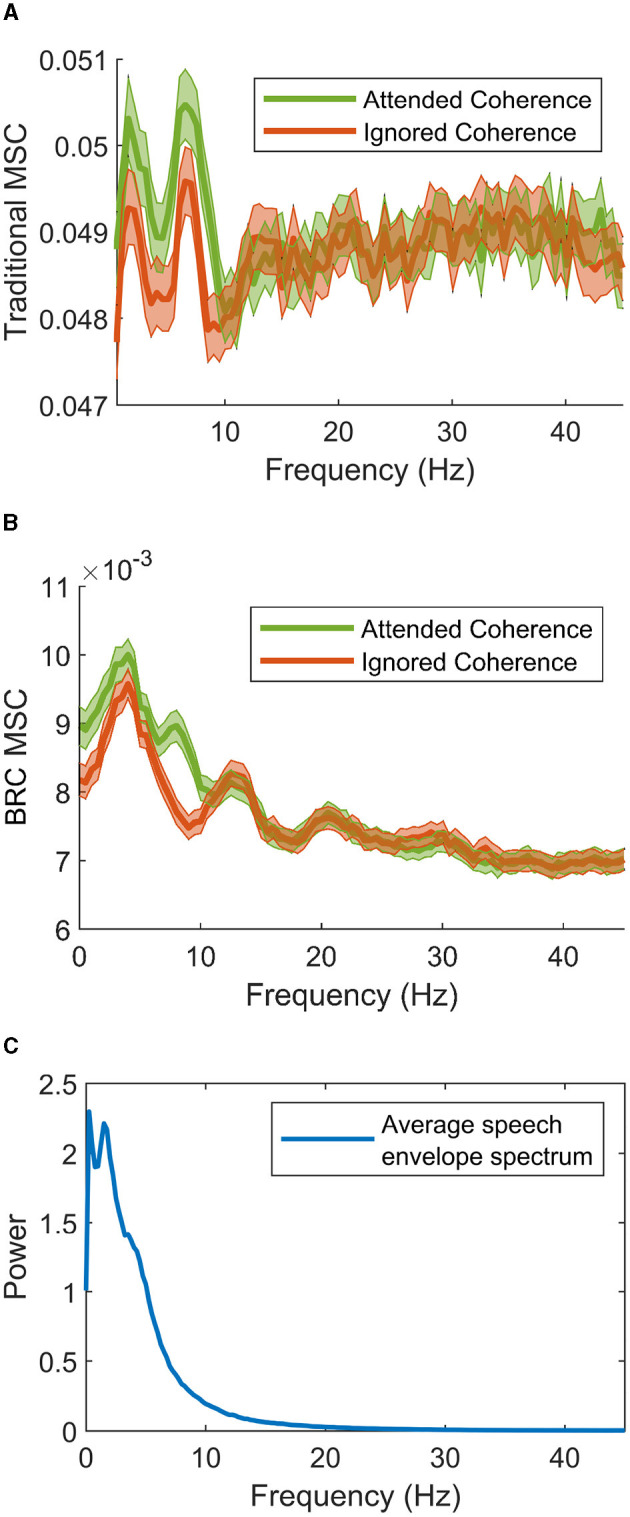
Grand averages of speech-EEG coherence between attended (green) and ignored (red) speech envelopes estimated using **(A)** the traditional method and **(B)** the BRC method. Colored areas show the empirical 95% confidence interval of estimates across the spectral range. Note the rather large difference in overall absolute value of estimates between the two methods on the *y*-axis. The average power spectra of all speech envelopes (both attended and ignored) is shown in **(C)**. One can observe a general correspondence in the shape of the spectra, comparing **(A, B)** to **(C)**.

To start, Figures 4A, B show the grand average of coherence estimates using the traditional method and BRC, respectively. These are shown beside the estimated power spectra of speech envelopes, averaged over all speech material, in Figure 4C. A general correspondence is found between the shapes of the different spectra, which is expected since the spectra are all based on the Fourier transform of speech envelopes. Both methods successfully differentiate attended and ignored speech, which is expected due to the success of linear filters in discriminating speakers in multi-talker situations for hearing impaired listeners. Notably, BRC provides less noisy coherence estimates across frequencies and exhibits much lower baseline coherence. The lower baseline coherence is a result of the reduced bias for low coherence estimates when using BRC, described in Keding et al. (2023). While both methods show a similar frequency pattern, the traditional method shows two clear coherence peaks, whereas BRC features one more pronounced peak at 3–5 Hz. The BRC captures a smaller peak in the 6–9 Hz range for attended speech-EEG coherence, highlighting a difference between speech streams. In contrast, the traditional method lacks this clear pattern as it results in peaks for both attended and ignored speech.

**Figure 5:**
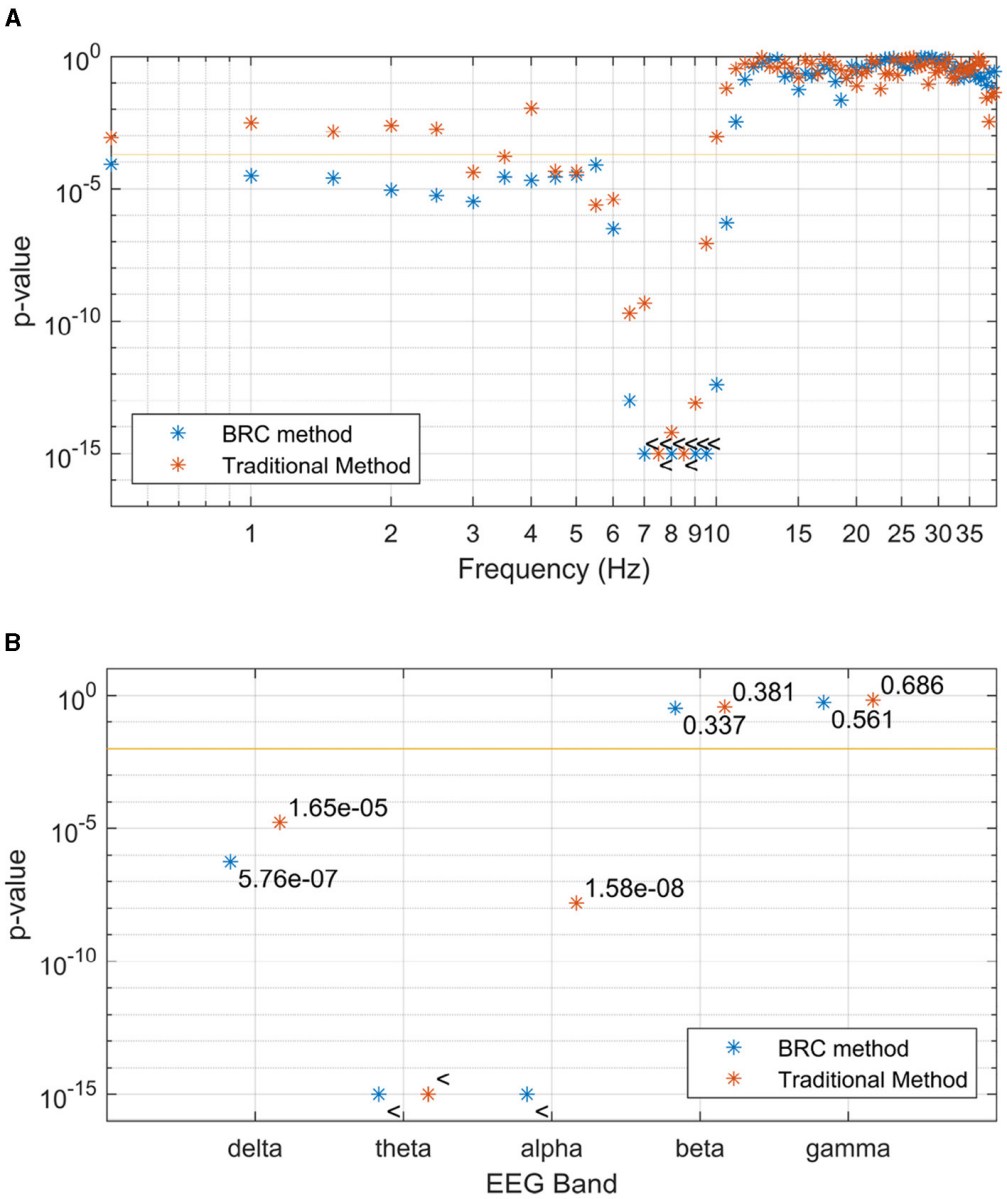
*P*-values, indicating the difference between observed speech-EEG coherence estimates for attended and ignored speech envelopes, are shown for **(A)** each frequency bin and **(B)** each EEG band. Traditional method-derived values are indicated in red, while the values in blue are computed using the BRC method. BRC consistently yields lower *p*-values, facilitating stronger statistical testing with reduced data requirements. With a few frequency bins as an exception, this is true for the frequencies and bands where *p*-values for any method are significant. The 95% significance level of *p*-values is marked by the horizontal line, with the < symbol indicating *p*-values below the cutoff.

The difference in grand averages seen in Figure 4 becomes more apparent through grand-level mean testing of the coherence. As detailed in Section 3.3, this approach provides three ways for assessing the significance of speech-EEG coherence differences between attended and ignored speech: at the band level, frequency bin level, and channel level. Figure 5A displays *p*-values, which evaluate speech-EEG difference between attended and ignored speech, for each frequency bin. The figure is cut at 40 Hz, but the pattern of non-significant *p*-values seen in the range of 10–40 Hz continues upwards in the frequency range above 40 Hz, omitted to enlarge the differences in *p*-values seen at lower frequencies. Figure 5B shows the significance of coherence differences when coherence estimates are averaged within each EEG band.

**Figure 6:**
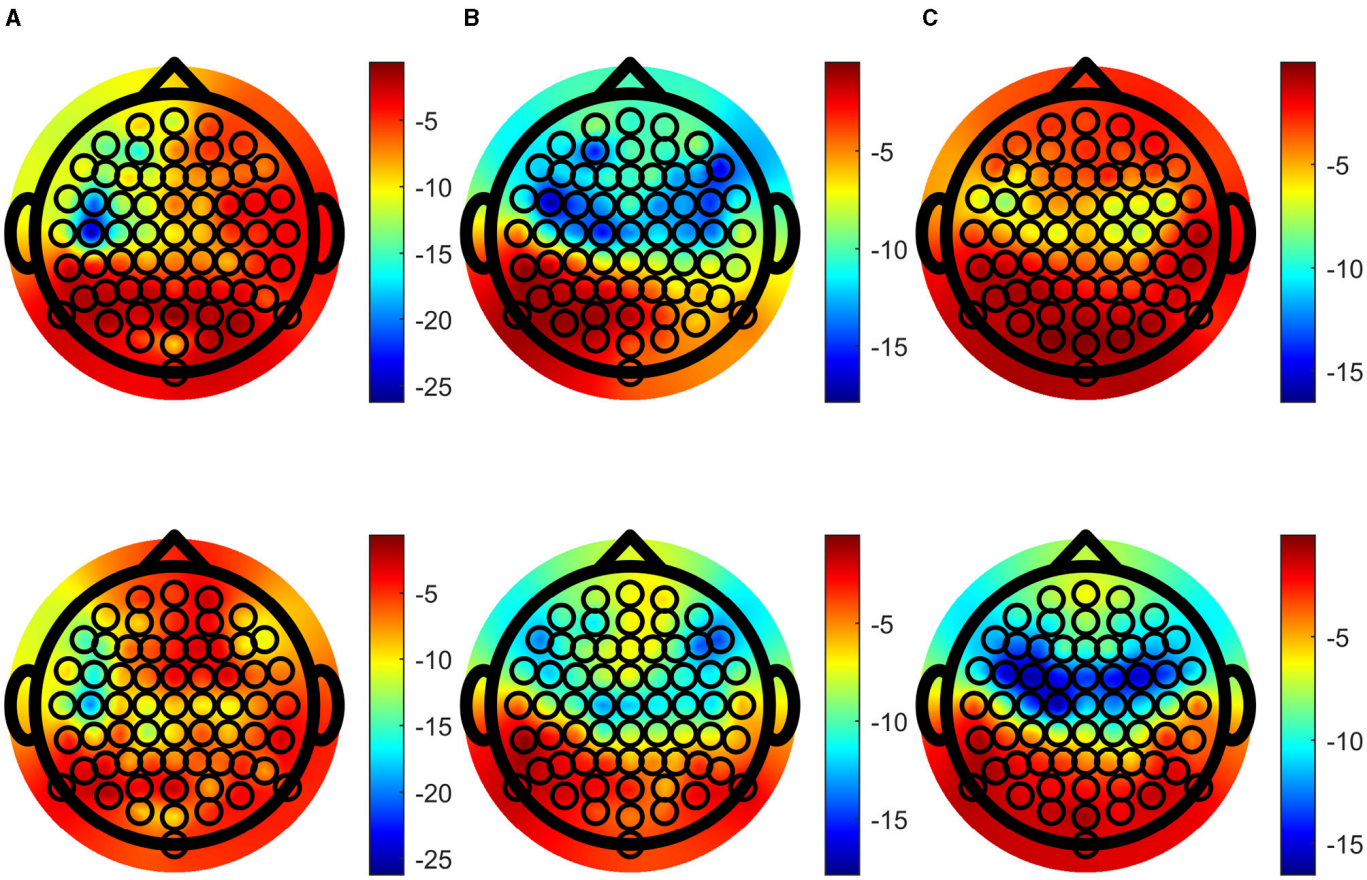
Topographic plots of the logarithm of *p*-values showing significant difference between attended and ignored coherence, **(A)** the delta band, **(B)** the theta band and **(C)** the alpha band. Upper figures show log *p*-values calculated with the traditional method, and lower figures are calculated with the BRC method. Color intensity of log *p*-values is matched over each column for comparison.

Speech-EEG coherence estimates for the delta, theta and alpha bands across each EEG channel were extracted and the grand averages of these were tested, resulting in topographic plots of logarithms of corresponding *p*-values, shown in Figure 6. Importantly, enhanced speech-EEG coherence for attended speech envelope compared to ignored speech envelope is more significant when *p*-values and logarithm of *p*-values are lower, as depicted by blue shading in the figure.

**Figure 7:**
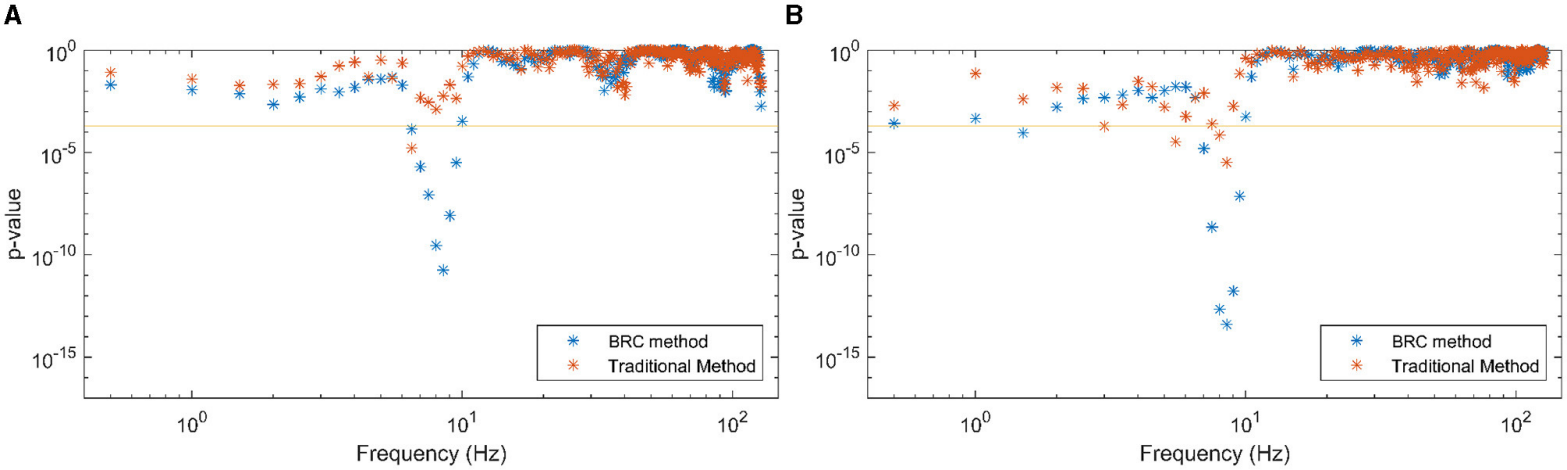
*P*-values, indicating the difference between observed speech-EEG coherence estimates for attended and ignored speech envelopes, are shown for **(A)** each frequency bin and **(B)** each EEG band. Traditional method-derived values are indicated in red, while the values in blue are computed using the BRC method. BRC consistently yields lower *p*-values, facilitating stronger statistical testing with reduced data requirements. The 95% significance level of *p*-values is marked by the horizontal line, with the < symbol indicating *p*-values below the cutoff. **(A)** Frequencies, HA NR OFF. **(B)** Frequencies, HA NR ON.

As a final piece of analysis, the effects of HA NR on the coherence measures of attended and ignored speech were investigated. Half of the trials involved participants with the NR algorithm OFF in their HAs, while the other half had it ON. After splitting the trials into conditions with HA NR OFF and HA NR ON and conducting identical statistical analysis of attended/ignored speech-EEG coherence differences (now with half the total number of trials for each condition), the resulting *p*-values are shown in Figures 7A, B. *P*-values are generally lower when HA NR is ON in frequencies showing a linear relationship between speech envelopes and EEG stronger than the same for when HA NR is OFF.

## 5 Discussion

The potential of the coherence measure for neural tracking of speech, compared to commonly used methods, is discussed in Section 5.1. In this section, discussion around the results of the BRC and traditional coherence method is also provided. Furthermore, Section 5.2 provides promising results that highlight speech-EEG coherence as a valuable objective measure for assessing the effects of HA signal processing algorithms on auditory attention.

### 5.1 Separating attended and ignored speech

As an initial discussion to motivate the use of coherence methods, we compare characteristics of the coherence methodology to the state-of-the-art TRF analysis paradigm. Commonly within the field of neural tracking of sound, a TRF filter is trained on attended speech, and then applied on other trials to distinguish between attended and ignored speech on these new trials. Given a certain decision window on unseen data, the previous fitting of TRF on other data will make the method better at classifying speech as attended or ignored, although this requires the fitting of the filter on previous data. The methodology of analyzing the difference in attended and ignored coherence, is algorithmically simpler. This approach requires no training or data splitting (train-test or cross-validation), and can be applied directly to new data to make conclusions about auditory attention. Additionally, in the fitting of filters, a method and level of regularization are required to be chosen. This problem, of choosing regularization hyperparameters, is avoided with the coherence methodology. Instead, hyperparameters are chosen during coherence estimation on new data. These hyperparameters can be deduced from the signal characteristics of speech features and EEG responses, to a very large extent. Therefore, coherence can be suitable for early screening of data where no training data is available, and data splitting is not possible.

Furthermore, as the coherence measure is calculated over each channel and frequency, analysis in these domains is straightforward. Comparatively, using TRF requires training and test of individual filters for each channel. Certain regularization techniques can also smear which frequencies are important in the filters. If classification between attended and ignored speech is of main interest, then coherence measures presented in this work can be used in further analysis to make classification models. In this work, the difference of coherence in frequencies and channels is used to differentiate between attended and ignored speech, although one can just as well use coherence as inputs to more complicated models.

With comments about the role of coherence in neural tracking of speech, interesting observations can be made when applying these methods. Initially, one can observe in Figure 4 that the coherence measures differ between are at the lower end of spectral axis, where also speech envelope power is strong (seen in Figure 4C). This is reasonable because it is the lower frequencies of the speech envelopes that carry information. High oscillations of envelopes are typically not informative for the speech envelope, as these not as prevalent.

For the statistical analysis of *p*-values in the frequency domain, shown in Figures 5A, B, two main observations can be made. Firstly, significant differences in coherence are observed in the delta, theta and alpha bands. Secondly, within the delta band, multiple individual bins exhibit significance with the new coherence estimation method BRC. This is particularly relevant, since the delta band should indeed exhibit a significant difference between attended and ignored speech envelopes, when estimated using linear filter methods, making BRC superior in regard to sensitivity, compared to the traditional approach (Bröhl and Kayser, 2021; McHaney et al., 2021). It is important to note that BRC and traditional coherence methods yield similar (not significant) *p*-values for frequencies and bands where a linear relationship between speech envelope and EEG is not anticipated. Also, considering the bias described in Section 2.2 and visualized in Figure 3, frequency bins showing coherence contains significant information from lower frequencies as the coherence peaks are shifted toward higher frequencies in slanted noise of EEG.

Focusing on the spatial patterns of coherence, Figures 6B, C strengthen the evidence for coherence differences in central and frontal regions, consistent with previous research (Fiedler et al., 2019; Lesenfants and Francart, 2020; Schmitt et al., 2022). *p*-values remain low across the parietal channels, in line with prior studies (Shahsavari Baboukani et al., 2022). Figures 6B, C also illustrate patterns where BRC results in smaller jumps in significance between neighboring channels, compared to the traditional one. The traditional method has more sensitivity over frontal and central channels in the theta band, and BRC has more sensitivity in the alpha band. Although it may be hard to make strong claims in regard to which alternative is preferable, each band include an equal amount of frequency bins. The significance gain in the alpha band using BRC is larger than the gain in the theta band using the traditional coherence method.

### 5.2 Coherence as an objective evaluation of HAs

This work suggests using coherence methods to objectively evaluate benefits of signal processing algorithms in HAs in real-life, multi-talker environments. As seen in Figures 7A, B, the *p*-values for the ON condition are lower than the *p*-values for the OFF condition. This suggests a more pronounced distinction between the coherence associated with attended speech compared to coherence associated with ignored speech. It implies that activating HA NR algorithm enhances the separation of attended speech from irrelevant sounds, thus aiding in the auditory attention task. This is corroborated by previous studies (Alickovic et al., 2020, 2021).

While beyond the scope of this study, a future research could explore the link between behavioral outcomes and coherence estimates in context of HA signal processing algorithms. Such an investigation could strengthen the proposed objective measure introduced for evaluating the benefits of HA technology. By delving into channels and frequencies of interest, it may demonstrate sensitivity of HAs with less EEG data. This could further facilitate clinical adoption of coherence measures as an objective assessment tool.

## 6 Conclusions

Linear system analysis has previously proven useful for inferring about the connection between speech features, in specific speech envelopes, and measured EEG representing cortical responses. This paper aligns with previous work, and show the functionality of using coherence to decode attention in a cocktail-party environment. Although using higher-order models is useful in their own right, the use of linear models to understand the brain is of importance since their effects on results can be easily quantified.

When interpreting brain electrical activity through spectral aspects of coherence, it is crucial to address two types of biases. Firstly, an upward bias toward higher coherence is analyzed due to responses having a very weak linear connection, showing an improvement with BRC. Moreover, this bias is mitigated by including more trials. Secondly, and of particular significance in EEG applications, there is the shifting of coherence peaks in spectral domain toward higher frequencies. This peak shifting is amplified when widening the effective bandwidth of the spectral estimation technique.

Finally, a novel coherence method, namely BRC, was introduced for objectively evaluating the efficacy of HA noise reduction algorithms in realistic listening environments. Observable differences in speech-EEG coherence between attended and ignored speech were observed when the noise reduction feature in HAs was activated compared to when it was deactivated. While no rigorous test is presented, the speech-EEG coherence difference between noise reduction schemes remains consistent over frequencies related to the listening task. Further work is needed to explore the suitability of coherence measures for objective evaluation of HAs, where parameters such as data amount are taken into account.

## Data Availability

The data analyzed in this study is subject to the following licenses/restrictions: due to this regulation and the way data was collected with low number of participants, it is not possible to fully anonymize the dataset and hence cannot be shared. Requests to access these datasets should be directed to Claus Nielsen, clni@eriksholm.com.
